# Effect of Silica Fume as a Component of Alternative Binder on the Selected Technically Important Characteristics of Bio-Aggregate-Based Composites

**DOI:** 10.3390/ma11112153

**Published:** 2018-11-01

**Authors:** Nadezda Stevulova, Jozef Junak, Vojtech Vaclavik

**Affiliations:** 1 Department of Material Engineering/Institute of Environmental Engineering, Faculty of Civil Engineering, Technical University of Kosice; Vysokoskolska 4, 042 00 Kosice, Slovakia; jozef.junak@tuke.sk; 2 Department of Environmental Engineering and Institute of Clean Technologies for Extraction and Utilization of Energy Resources, Faculty of Mining and Geology, VSB-Technical University of Ostrava; 17. Listopadu 2172/15, 708 33 Ostrava—Poruba, Czech Republic; vojtech.vaclavik@vsb.cz

**Keywords:** bio-aggregate-based composite, alternative binder, compressive strength

## Abstract

This experimental study was focused on the application of an alternative binder based on MgO, and the variation of its components by the combination of two MgO products obtained by the calcinations of natural magnesite, siliceous materials (river sand and silica fume), and alkaline admixture in the mixture for a preparation of composite based on biomass waste such as hemp hurds as organic filler. This paper presents the results of the effect of an MgO binder composition on the compressive strength of the bio-aggregate-based composites. Other physical properties, such as the bulk density, thermal conductivity coefficient, and water absorption, were also investigated. The measured strength parameters of the bio-composite samples that were hardened for 28 days demonstrate that the binder consisting of optimal calcined MgO and silica fume as a total replacement for sand ensures a good binding of the matrix with hemp hurd compared to other varied compositions of alternative binder. No significant differences in bulk density and thermal conductivity values were found for these composites. However, the bio-composite specimen with an MgO–SiO_2_ matrix had the highest compressive strength and achieved the lowest value of water absorption. An increase in hardening time of up to 90 days led to a significant improvement of strength as well as reduction in permeability.

## 1. Introduction

Environmentally-friendly properties of lightweight composites based on hemp hurds have attracted the attention of researchers around the world [1,2,3,4,5,6,7,8,9]. Hemp hurds, as a cellulose material obtained from the woody core of the hemp plant (*Cannabis Sativa* L.), are used in the preparation of building materials, and mainly in the production of thermal insulation composites, due to their porous structure and low density. The current trend in civil engineering is devoted to composites based on hemp hurds with an inorganic matrix [6,9,10]. Portland cement, as a popular inorganic hydraulic binder, is a key ingredient of concrete structures. As shown in papers by Taylor et al. and Moya et al. [11,12], the global demand for cement and concrete will continue to rise during the next 40 years. At present, the production of cement as a traditionally used binder is accompanied by environmental problems. Moreover, natural fibrous cellulose material in cement-bonded composites undergoes degradation owing to its decomposition in an alkaline environment, leading to a reduction of fiber–matrix adhesion and subsequently to a strength decrease of the whole composite [13,14]. The long durability of bio-based composites may be also limited by the mineralization of internal pore walls in the fibrous particles due to the deposition of hydration products. To avoid the problem of the limited durability of cellulose material in a cement matrix with the a pH range of 12.7–13.9 (depending on the alkalinity of the cement binder), attention is being given to modify the cement matrix by using various supplementary cementitious materials, as well as alternative binders, ensuring a matrix with lower alkalinity [15]. Many studies have been carried out to replace Portland cement in composites based on natural cellulosic fibers and bio-based aggregates of lignocellulosic waste by alternative binding materials. The aims of such work are to reduce the environmental footprint and costs associated with the cement production, enhance the physical and mechanical properties of the composite, and prepare innovative and more sustainable building materials. Industrial wastes and by-products, such as stone quarry dust, saw dust, fly ash, blast furnace slag, reservoir sediments, glass, ceramic wastes, and silica fume are appropriate alternatives, as they contain the important cementitious substances, such as calcareous and siliceous components [16,17,18,19,20,21,22,23]. The pozzolanic properties and availability of these wastes predetermine their utilization in preparing building materials. The effects of various parameters, such as the binder nature and chemical composition, plant origin, mean particle length, and fiber treatment method, on the mechanical properties of bio-composites have been investigated [24,25,26,27,28,29,30]. Numerous binders may be used to make building materials based on aggregates of plant origin [31]. In Europe, the most used binder among alternative binding materials is hydraulic/hydrated lime, in combination with cement or lime itself as a reactant with a high specific surface area. However, the lime hydration is affected by the water-soluble sugars that are present on the surface of hemp hurd particles [32]. Pectin and other partially soluble components of the hemp structure react with Ca^2+^ ions in lime, resulting in less calcium ions available for the formation of hydration products [33]. Due to this phenomenon, values of compressive strength of so-called hemp concrete were found to be too low (about 1–2 kPa) for use in structural purposes as a load-bearing material [34]. The standard method of applying hemp concrete in building construction is using a wooden frame; the plywood shuttering of walls associated with a frame are filled with hemp concrete.

The comprehensive efforts made in our previous studies were focused on a partial/total cement replacement by using hydrated lime [15], natural zeolite, and MgO binder [35,36] in building composites based on bio-aggregates of hemp hurds. MgO binder as a clinker-free binder, consisting of calcined magnesite, siliceous sand, and alkaline additive, in combination with cellulose filler, provided composite material with an improved strength parameter after 28 days, 60 days, and 90 days of hardening in comparison to cement-based composites [37]. Compressive strength values were higher by 14–32% than those of the Portland cement composite at all curing times. Hemp hurds composites prepared with MgO binder after 90 days of hardening achieved compressive strength values that were 2.5 times higher (close to 5 MPa) compared to those achieved after 28 days [38]. For these reasons, our research initiatives were oriented toward the optimization of a mix design based on MgO binder, following the current trend of focusing on the use of alternative materials in MgO–SiO_2_ systems. Aiming at improving the properties of bio-aggregate composites with alternative calcium-free binders, this work has investigated the effect of designing an MgO binder composition on the physical and mechanical properties of 28-day-hardened hemp hurd composites. This paper presents the compressive strength test results and other physical characteristics of hemp hurd composites where the crystalline sand in MgO binder was replaced by silica fume in different proportions. The goal was to assess the effect of the MgO binder composition on the properties of the bio-aggregate-based composites.

## 2. Materials and Methods

### 2.1. Material

The technical hemp hurds used in this study as filler in composites were acquired from Hemp Flax (Oude Pekela, The Netherlands). This bio-material came from the plant stem of Cannabis sativa L. cultivated for the fibers or seeds. Bio-based aggregates of hemp hurds are a lightweight waste material (density of 117.5 kg·m^−3^) from processing hemp into fibers. The material that was used in the study consisted of a large majority of hemp hurds over hemp bast fibers [36]. The particle size distribution of the hemp hurds slices was characterized by a wide range of sizes (0.063–8 mm). Fine dust particles originating from the manufacturing disintegration process were also contained in hurds. The particle size of hemp particles was evaluated by their length, because the bio-aggregates were several times longer than their diameter. A mean length of hemp hurds of 1.94 mm was calculated. The average moisture of hemp hurds, as a highly water-absorbing substance, was determined to be close to 11%. According to the results of the chemical analysis of hemp hurds described in our previous paper [39], cellulose, hemicelluloses, and lignin are the main components of this fibrous material. The content of non-cellulosic components such as lignin, waxes, and ash, was about one-third the amount of holocellulose.

**Figure 1:**
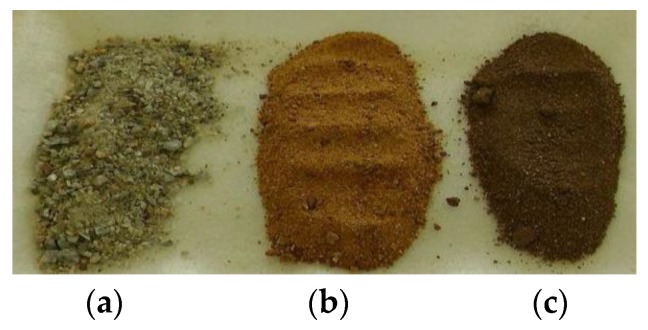
Comparison of samples: natural magnesite (**a**), MgO–L (**b**) and MgO–C (**c**) of the mean particle diameter of 1031 μm, 385 μm, and 337 μm.

The MgO binder consisted of three components: MgO obtained from the calcination of magnesite, silica sand, and an alkaline admixture of sodium hydrogen carbonate (NaHCO_3_) in a 1:1:1 ratio. The alkaline substance (A) was a high-purity chemical (p.a.) supplied by Gavax (Vranov nad Toplou, Slovakia). Two kinds of MgO were used: commercial product CCM 85 calcined at 850 °C (MgO–C) from Slovak Magnesite Factory (Jelsava, Slovakia), and a sample prepared by the calcination of Jelsava magnesite of the breunerite type in a ceramic high-temperature furnace FERRMAT KO 2 El.re (Ferrmat, Kosice, Slovakia) at an optimal temperature of 700 °C (MgO–L). Figure 1 illustrates the changes in the color of natural magnesite raw material in comparison to the used MgO products. These color differences are due to changes in the chemical composition during the thermal decomposition of magnesite when carbon dioxide molecules are released from the crystalline carbonate structure, and this is accompanied by decrepitating the particles. A decrease of the mean particle diameter was found (see Figure 1). Both magnesium oxide samples were milled in order to reduce their particle size in a laboratory vibratory mill VM 4 (Brio, Hranice, Czech Republic) [40] to several microns. The chemical composition and specific surface area of both MgO products is given in Table 1. An X-ray diffraction powder analysis of MgO products showed the presence of the crystalline phases of periclase, calcite, dolomite, and quartz. However, the quantity of the main crystalline phase (periclase) was low because of a prevailing röntgen amorphous phase. Other identified phases come from the mineral texture of natural magnesite with isomorphically embedded iron and calcium ions in its carbonate structure.

Two kinds of siliceous component in an alternative binder based on MgO were used: (1) certified river sand (RS) (Sastin, Slovakia) with the dominant component of SiO_2_ (95–98 wt.%); and (2) silica fume (SF), a sand replacement, as a by-product resulting from the production of metallic silicon or ferrous-silicon alloys containing at least 85 wt.% of amorphous silica (OFZ, Istebne, Slovakia). The granularity characterized by the mean particle diameter (calculated from the distribution particle size function) of these materials was different; SF was a powder with a particle size much finer than that of sand particles (0.85 μm and 386 μm, respectively).

Variations of the MgO–binder composition are given in Table 2.

### 2.2. Preparation of Hemp Hurd Composites

Seven experimental mixtures were prepared according to the recipe (40 vol. % hemp hurds, 29 vol. % binder, and 31 vol. % water) published by Bydzovsky in work [41]. Mixing of the components (hemp hurds and MgO–binder) was firstly carried out manually in a dry manner in a large vessel to achieve a homogenized mixture. Then, water was gradually added. A 50-L mixer was used for final stirring lasting 3 min. Standard steel cube molds with dimensions of 100 × 100 × 100 mm were gradually filled with the prepared mixture. Manual compaction in three layers was performed in a pestle. The inner mold surface was oiled to prevent sticking of the specimen and mold. The specimens of the fibrous composites in molds were cured for two days in an indoor climate (23 ± 1 °C, relative humidity of 60 ± 5%). After this time, the mold was removed, and specimens were covered with a plastic foil. The curing of specimens (with recipes of B0–B6) continued under laboratory conditions of fixed values of air temperature and relative humidity (as mentioned above) for 28 days. The mixture with complete sand substitution with SF (B7) was also subjected to hardening for 60 days and 90 days.

### 2.3. Testing Methods

**Figure 2:**
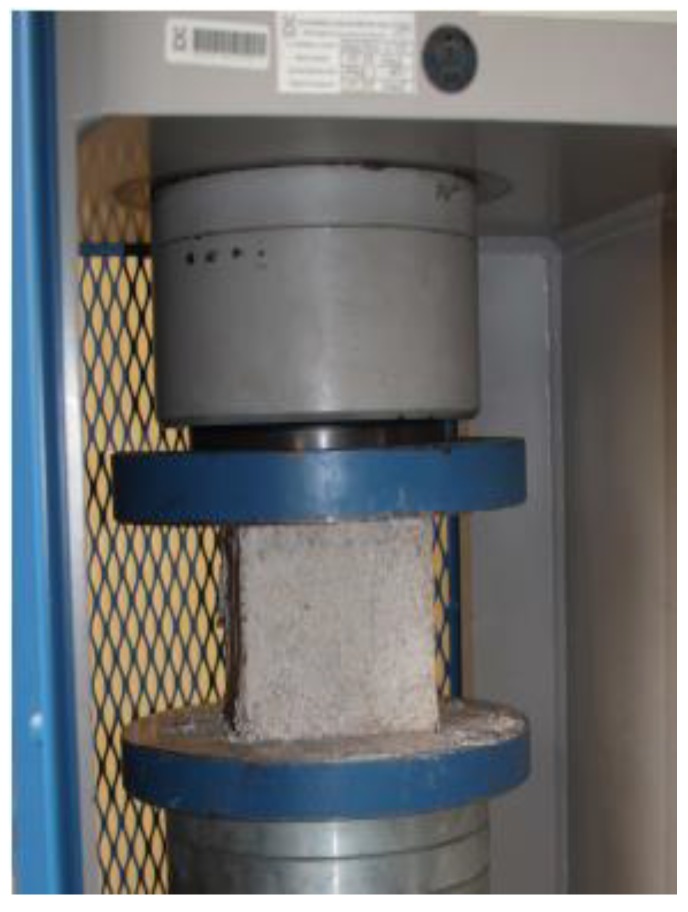
Test composite specimen in the device for determination of compressive strength.

The selected physical and mechanical properties of hardened specimens were tested under controlled laboratory conditions. Due to moisture elimination and the credibility of the tested parameters, the samples were dried in a ventilated oven at 60 °C to a constant weight. Compressive strength was determined under controlled conditions using a constant loading rate (0.3 MPa/mm^2^/s = 3.0 kN/mm^2^/s), as the maximum load per average cross-sectional area on the instrument ADR 2000 (ELE International Ltd., Sheffield, United Kingdom) (Figure 2) in accordance with the standard [42]. The tests were performed in triplicate, and the average value was reported. The bulk density and thermal conductivity of hardened composites were also investigated. Bulk density was determined in accordance with standard STN EN 12390-7 [43]. The thermal conductivity coefficient of samples, as the main parameter of heat transport, was measured using the commercial device ISOMET 104 (Applied Precision Ltd., Bratislava, Slovakia) at selected work points on each side of the cube. The resulting value of this parameter was expressed as the average value of 18 measurements. A short-term water absorption test was performed on these specimens by immersion in water for 1 h at 23 ± 1 °C using the method of determining long-term water absorption specified in the standard [44].

## 3. Results and Discussion

The selected physical properties (bulk density, thermal conductivity coefficient, and short-term water absorption) and mechanical properties, represented by compressive strength of 28-day hardened hemp hurds composite samples with a variety of MgO binder compositions, were measured. As can be seen from Table 3, the bulk density, thermal conductivity coefficient, water absorption, and compressive strength values of these composite samples are dependent on MgO binder composition. The deviation of the measured values from the average value of the individual parameter varied around 5%.

Based on the density range (760 kg/m^3^ to 920 kg/m^3^) of these materials in a dry state, they can be included in the group of lightweight composites in which the bulk density ranges from 200 kg/m^3^ to 2000 kg/m^3^. The obtained bulk densities of these composites are lower than those composites of the same formulation (B0), but are prepared with chemically treated hemp hurds [36].

The thermal conductivity coefficient values of the prepared bio-aggregate specimens with MgO binder changed in a narrow range from 0.170 W/m·K to 0.205 W/m·K. These measured values of hemp hurd composite specimens based on MgO binder are more favorable when compared to the values of the conductivity parameter obtained for hemp composites with a lime binder and Portland cement [45]. These thermal conductivities correspond to the lower values of the conductivity parameter range for the lightweight concrete (0.11–1.25 W/m·K); however, they are lower than the value characterizing the thermal insulating material (0.3W W/m·K). This confirms that all hemp hurd composite specimens have a thermal insulating function.

As is clear from Table 3, the short-term water absorption of hardened hemp hurd composites is relatively high, with values ranging from 15.1% to 22.4%. Approximately the same value of short-term water absorption (around 15%) was achieved by two composite samples—MgO binder compositions B5 and B7—where MgO–L, siliceous component, and no alkaline admixture were used.

The mechanical performance of composites prepared using bio-aggregates of hemp hurds and MgO–binder was evaluated. The values of the compressive strength of all of the composites were in the range of 1.55–3.51 MPa. Composite B0 as a comparative sample prepared with MgO–C, RS, and alkaline admixture (A), achieved the lowest compressive strength. The studied strength parameter of all of the hemp hurd composites prepared from MgO–binders with added alkaline admixture (B2, B4, and B6) did not exceed 3 MPa, which is in contrast to the composite produced from the mixture of MgO–L and SF (B7). This combination of binder components (B7) led to the enhancement of mechanical properties of the hemp hurd composite specimen. Comparing the development of compressive strength values with the prolonged hardening time in Table 4 indicates the favorable increase in the strength parameter, after 90 d of hardening, up to 8.12 MPa. The combination of binder components (MgO–L and SF) makes the hemp hurd composite stronger and denser, and thus reduces its permeability, as evidenced by the short-term water absorption values (Table 4).

As is known, the compressive strength of bio-aggregate composites is determined by interfacial adhesion between particles of filler and binder. The reactivity of the starting materials of MgO and SiO_2_, and the degree of hydration are also key factors influencing the reaction process in the mixture. As shown in paper by Li et al. [46], the pH value of pore solution (about 10.5) in the MgO–SiO_2_–H_2_O system plays a crucial role in the formation of magnesium silicate hydrates (M–S–H). The amorphous M–S–H phase, having the properties of a rigid gel, is responsible for the binding characteristics between MgO and SiO_2_ components. However, a starting point for the development of M–S–H gel is the hydration of MgO. Whereas M–S–H formation in mixtures based on the original mix design of an alternative MgO binder is limited because of a high pH value (around 12.6) [47], binder components of MgO–L and SF, with their high specific surface areas (88.1 m^2^ g^−1^ and 31.9 m^2^ g^−1^, respectively), provide more favorable conditions for the hydration and formation of M–S–H. The lower pH value of the MgO–SiO_2_ system of around 10.7, which is represented by mix sample B7, could probably indicate the extent of hydration of the system, because SF itself reaches a pH value around 5.5 [48]. During the hydration process, the transition interfacial zone is gradually densified due to pozzolanic reactions between SF and MgO components. CaO, which is found in small quantities in the MgO-L product, is present only in carbonate form. As is evident from the presented results, an MgO–SiO_2_ matrix consisting of MgO calcined under conditions of optimal temperature (with the highest specific surface area value) and silica fume enhance the strength properties of the lightweight bio-aggregate composite. Our conclusions about a good adherence between M–S–H gel and cellulosic materials are in accordance with the interpretation by Mármol et al. in [13] where the use of a calcium-free cement system based on MgO and SiO_2_ in sisal fiber composite was investigated. The objective of subsequent research will be to study the chemical properties of hemp hurd aggregates incorporated in an MgO–SiO_2_ matrix and analyze the formed hydration products.

## 4. Conclusions

Based on the experimental results, the bulk density and thermal conductivity of 28-day hardened hemp hurd composites are not significantly affected by binder composition. On the contrary, water absorption and compressive strength depend on the variation and proportion of binder components in the mixture. The highest compressive strength value among 28-day hardened hemp hurd composites with varying binder composition was achieved by the specimen with binder components of MgO, as prepared by calcination of natural magnesite at an optimal temperature of 700 °C, and silica fume. This combination of components of high reactivity (high fineness and low degree of crystallinity) creates favorable conditions for M–S–H phase formation. An increase in hardening time by up to 90 days led to relatively high strength parameter values that were 2.3 times higher than those found for the 28-day composite. A reduction in the water absorption value of 40% was also found.

The obtained results have confirmed the positive role of an alternative MgO binder in bio-aggregate-based composites. The use of an alternative binder based on an MgO–SiO_2_ system could lead to development of an innovative bio-composite based on the lignocellulosic waste material of hemp hurds. This prepared composite shows good mechanical performance as a non-load bearing material, and has great potential as a material with good thermal insulation due to the low bulk density and porous structure of hemp hurd aggregates.

In order to understand the context related to the resulting properties of the bio-composites, further research will be focused on the detailed study of hydration products as well as a characterization of the changes in chemical properties of hemp hurds incorporated in this matrix. Aiming at increasing the reactivity of the MgO–SiO_2_ mixture, a specific focus will be given to the mechanochemical activation of this system.

## Figures and Tables

**Table 1:** Chemical composition of MgO products and specific surface area.

Oxide	Content (%)	
	MgO–C	MgO–L
MgO	84.0	69.1
CaO	5.5	5.2
Fe_2_O_3_	7.5	6.0
SiO_2_	1.0	0.6
Al_2_O_3_	0.2	0.08
Loss on ignition (%)	1.7	18.5
Specific surface area (m^2^·g^−1^)	47.0	88.1

**Table 2:** Components variation in MgO–binder (RS—river sand; SF—silica fume; A—alkaline admixture).

Binder Sample	Components Variation in Binder (%)				
	MgO–C	MgO–L	RS	SF	A
B0	33.3		33.3		33.3
B1	50		50		
B2	33.3			33.3	33.3
B3	50			50	
B4		33.3	33.3		33.3
B5		50	50		
B6		33.3		33.3	33.3
B7		50		50	

**Table 3:** Physical characteristics and compressive strength of 28-day hardened bio-composites.

Binder Sample	Density (kg/m^3^)	Thermal Conductivity Coefficient (W/m·K)	Shor-Term Water Absorption (%)	Compressive Strength (MPa)
B0	890 ± 4	0.195 ± 0.015	22.4 ± 1.05	1.55 ± 0.06
B1	910 ± 3	0.205 ± 0.014	19.8 ± 1.24	2.84 ± 0.16
B2	810 ± 3	0.180 ± 0.011	19.9 ± 0.97	2.40 ± 0.11
B3	790 ± 8	0.180 ± 0.011	16.1 ± 0.83	3.32 ± 0.21
B4	770 ± 7	0.170 ± 0.009	20.2 ± 1.06	1.75 ± 0.10
B5	920 ± 6	0.190 ± 0.018	15.1 ± 1.12	2.94 ± 0.19
B6	805 ± 5	0.175 ± 0.015	20.3 ± 1.02	2.76 ± 0.15
B7	760 ± 4	0.175 ± 0.011	15.3 ± 0.74	3.51 ± 0.17

**Table 4:** Development of compressive strength and short-term water absorption of bio-composite with binder composition of B7 in relation to hardening time.

Hardening Time (Days)	Short-Term Water Absorption (%)	Compressive Strength (MPa)
28	15.3 ± 0.74	3.51 ± 0.17
60	11.1 ± 0.53	4.59 ± 0.28
90	8.9 ± 0.41	8.12 ± 0.62
